# DeepNEU: Artificially Induced Stem Cell (aiPSC) and Differentiated Skeletal Muscle Cell (aiSkMC) Simulations of Infantile Onset POMPE Disease (IOPD) for Potential Biomarker Identification and Drug Discovery

**DOI:** 10.3389/fcell.2019.00325

**Published:** 2019-12-06

**Authors:** Sally Esmail, Wayne R. Danter

**Affiliations:** 123Genetix, London, ON, Canada

**Keywords:** iPSC, IOPD, deep machine learning, recurrent neural networks (RNN), cognitive maps (CM), support vector machines (SVM)

## Abstract

Infantile onset Pompe disease (IOPD) is a rare and lethal genetic disorder caused by the deletion of the acid alpha-glucosidase (GAA) gene. This gene encodes an essential lysosomal enzyme that converts glycogen to glucose. While enzyme replacement therapy helps some, our understanding of disease pathophysiology is limited. In this project we develop computer simulated stem cells (aiPSC) and differentiated skeletal muscle cells (aiSkMC) to empower IOPD research and drug discovery. Our Artificial Intelligence (AI) platform, DeepNEU v3.6 was used to generate aiPSC and aiSkMC simulations with and without GAA expression. These simulations were validated using peer reviewed results from the recent literature. Once the aiSkMC simulations (IOPD and WT) were validated they were used to evaluate calcium homeostasis and mitochondrial function in IOPD. Lastly, we used aiSkMC IOPD simulations to identify known and novel biomarkers and potential therapeutic targets. The aiSkMC simulations of IOPD correctly predicted genotypic and phenotypic features that were reported in recent literature. The probability that these features were accurately predicted by chance alone using the binomial test is 0.0025. The aiSkMC IOPD simulation correctly identified L-type calcium channels (VDCC) as a biomarker and confirmed the positive effects of calcium channel blockade (CCB) on calcium homeostasis and mitochondrial function. These published data were extended by the aiSkMC simulations to identify calpain(s) as a novel potential biomarker and therapeutic target for IOPD. This is the first time that computer simulations of iPSC and differentiated skeletal muscle cells have been used to study IOPD. The simulations are robust and accurate based on available published literature. We also demonstrated that the IOPD simulations can be used for potential biomarker identification leading to targeted drug discovery. We will continue to explore the potential for calpain inhibitors with and without CCB as effective therapy for IOPD.

## Introduction

Infantile onset Pompe disease (IOPD) (OMIM 232300) is a rare but lethal glycogen storage disorder caused by either loss or reduced activity of the lysosomal enzyme acid alpha-1,4-glucosidase (GAA, EC 3.2.1.20). This ubiquitously expressed enzyme is responsible for the conversion of glycogen to glucose (Hers, 1963). GAA deficiency leads to glycogen accumulation in lysosomes and the cytoplasm, leading to compromised tissue function. Skeletal muscle and cardiac tissue are most commonly affected by GAA deficiency (Hers, 1963). IOPD is rare with incidence estimate of 5000–10000 worldwide. As IOPD is a rare disease, diagnosis is often challenging, leading to delays and inaccurate estimation of disease severity (Dasouki et al., 2014). To date, more than 450 mutations have been identified in the GAA gene. IOPD affected infants present with a variety of abnormalities including loss of muscle tone, abnormal electrocardiogram, myocardial thickening, generalized weakness and hepatomegaly^1^.

IPOD patients rarely survive beyond 1 year of age without treatment. Enzyme replacement therapy (ERT) with recombinant human GAA (rhGAA) can be effective in symptomatic patients with cardiac abnormalities, but its effect on skeletal muscle symptoms is limited.

Thus, the development of novel therapeutic approaches to IOPD is urgently needed. The mechanism of skeletal muscle damage in IOPD disease has not yet been fully elucidated. A former explanation for the muscle damage was proposed to be the result of lysosomal rupture due to glycogen accumulation and the release of its lytic enzymes into the cytoplasm (Griffin, 1984; Thurberg et al., 2006). More recent studies evaluating GAA knockout mice or muscle biopsies from patients with IOPD suggested that secondary autophagic dysfunction plays an important role in progressive muscle damage (Fukuda et al., 2006a,b,c; Raben et al., 2007, 2010). However, in another study such autophagic dysfunction was not remarkable in the muscle of patients with IOPD despite the extremely enlarged lysosomes (Tiscornia et al., 2011), suggesting the possibility of a different mechanism of muscle damage in IOPD or GAA knockout mice.

Human induced pluripotent stem cells (iPSCs) are powerful tools for disease modeling due to their potential for differentiation into various types of tissue (Huang et al., 2011). Several disease models using patient iPSCs have been reported previously (Novak, 2006b; Sato et al., 2015, 2017). Recently an efficient iPSC-based skeletal muscle model of IOPD has been established to address some of the unsolved clinical problems described above (Sato et al., 2015, 2017). Despite the advances in IOPD research, many questions still require answers using a thorough systematic approach.

A biomarker is a gene, protein/peptide or metabolite present in a biological system that can be used to identify a physiological or pathological state that can be recognized or monitored (Young, 2000; Goymer, 2006; Novak, 2006a; Danter, 2019). Biomarker discovery is a challenging process; a good biomarker must be sensitive, specific and its test highly standardized and reproducible. The introduction and advances in computational technologies for disease modeling and biomarker discovery permit systematic approaches to biological discovery, which will have a profound impact on biological research, pharmacology, and medicine. The ability to obtain quantitative information about the complete transcription profile of cells promises to be an exceptionally powerful means to explore basic biology, diagnose disease, facilitate drug development, tailor therapeutics to specific pathologies, and generate databases with information about living processes and molecular mechanism of disease (Young et al., 2012; Yoshida et al., 2017; Elitt et al., 2018).

In 1956 the term Artificial Intelligence (AI) was defined by John McCarthy as “the science and engineering of making intelligent machines” (McCarthy, 1956). Since then an important subset of AI called Machine Learning (ML) has emerged at the forefront of AI research. Samuel (1967) described ML as “a field of study that gives the ability to the computer for self-learn without being explicitly programed.” The current study of ML continues to evolve rapidly. It strives to achieve inference over deduction and relies on statistical models, an assumption free (stochastic) approach to big data and constantly evolving learning algorithms.

In our recently published research, we have introduced DeepNEU, a novel unsupervised deep-machine learning computational platform that enables the successful generation of artificially induced pluripotent stem cells (aiPSCs), artificially induced neural stem cells (aiNSCs) and artificially induced cardiomyocytes (aiCMCs) (Danter, 2019). The DeepNEU platform is a validated hybrid deep-machine learning system with elements of fully connected recurrent neural networks (RNN), cognitive maps (CM), Support Vector Machines (SVM) and evolutionary systems. The detailed methodology for model development employed in the current study has been described previously in Danter (2019) and is detailed in the Supplementary Material (Appendix 1).

In this study, we have generated artificially induced IOPD pluripotent stem cell (aiPSC) and differentiated skeletal muscle cell (aiSkMC) simulations of IOPD to extend and empower further research and targeted drug discovery. The initial goal of this project was to first generate aiPSC and aiSkMC simulations with and without deletion of GAA, and then validate those models using the results published by Yoshida et al. (2017) and others. Once the aiSkMC simulations (WT and IOPD) were validated they were used to evaluate calcium homeostasis and mitochondrial function in IOPD. Finally, we extended this research to identify novel biomarkers and potential therapeutic targets specific for IOPD.

## Materials and Methods

The DeepNEU platform is a validated hybrid deep-machine learning system with elements of fully connected recurrent neural networks (RNN), cognitive maps (CM) and evolutionary systems. The detailed methodology for model development employed in the current experiments has been described elsewhere (Danter, 2019) and is also enclosed as Appendix 1. The current DeepNEU database (v3.6) contains the information found in the previous version (v3.2) plus an important information upgrade in the form of phenotypic concepts. For example, phosphorylation status of proteins like AKT, mTOR, S6K, Caspase 9 and numerous others are now encoded. Other examples of phenotypic concepts include dementia, neurodegeneration, seizure activity, reactive oxygen species (ROS), mitochondrial dysfunction, RBC hemolysis and hundreds of additional concepts. Each phenotypic concept has ∼8 gene/protein or phenotypic inputs and outputs.

### The DeepNEU Simulations

The initial goal of this project was to first create a computer simulation (aiPSC) of a human induced pluripotent stem cell (iPSC) and skeletal muscle cell models of Infantile onset POMPE disease (IOPD) and then validate these models using the results published by Yoshida et al. (2017) and others as described above. Briefly, for the aiPSC model the input or initial state vector of dimension N was set to all zeros except for transcription factors OCT3/4, SOX2, KLF4 and cMYC. These four factors were given a value of +1 indicating that they were turned on for the first iteration. These values were not locked on so that all subsequent values were determined by evolving system behavior. The skeletal muscle models (aiSkMC) employed direct conversion of fibroblasts to skeletal muscle cells by overexpression of MYOD1 in the presence of Doxycycline. The four models generated in the present study are summarized in Table 1 below.

**TABLE 1:** Summary of evaluated study models.

**Model**	**Status**	**Recipe**
aiPSC (WT)	Pluripotent	Fibroblast + OKSM + Dox + MYOD1
aiPSC (IOPD)	Pluripotent	Fibroblast + OKSM + Dox + MYOD1 – GAA
aiSkMC (WT)	Differentiated	Fibroblast + MYOD1 + Dox
aiSkMC (IOPD)	Differentiated	Fibroblast + MYOD1 + Dox – GAA

*OKSM, OCT3/4, KLF4, SOX2, cMYC; GAA, acid alpha-glucosidase; Dox, Doxycycline.*

The final predictions from the aiPSC and differentiated models regarding the expression or repression of genes and proteins and presence or absence of phenotypic features were directly compared with published data (Yoshida et al., 2017). Model prediction values ≥ 0 were classified as expressed or upregulated for genes/proteins or present in the case of phenotypic features while values < 0 were classified as downregulated, not expressed, or absent.

Statistical analysis of the aiPSC predictions and the published data used the unbiased binomial test. This test provides an exact probability, can compensate for prediction bias and is ideal for determining the statistical significance of experimental deviations from an actual distribution of observations that fall into two outcome categories (e.g., agree vs. disagree). A *p*-value < 0.05 is considered significant and is interpreted to indicate that the observed relationship between aiPSC predictions and actual outcomes is unlikely to have occurred by chance alone.

### DeepNEU Platform Specification

The DeepNEU database (Version 3.6) contains 3781 gene/proteins or phenotypic concepts and 31,027 non-zero relationships resulting in a large amount of information flowing into and out of each node in the network. On average, each node in the network initially has ∼8 inputs and 8 outputs. An analysis of all positive and negative network connections revealed a bias toward positive outputs. The pretest probability of a positive outcome prediction is 0.66 and the pretest probability of a negative prediction is therefore 0.34. This system bias was used when applying the binomial test to all simulation outcomes.

## Results

### The aiPSC (WT) Simulations

Previously published data have shown that iPSCs express many factors that are consistent with the signature of undifferentiated human embryonic stem cells (ESC). These factors include, OCT3/4, SOX2, NANOG, growth and differentiation factor 3 (GDF3), reduced expression 1 (REX1), fibroblast growth factor 4 (FGF4), embryonic cell-specific gene 1 (ESG1/DPPA5), developmental pluripotency-associated 2 (DPPA2), DPPA4, and telomerase reverse transcriptase (hTERT) (Adewumi et al., 2007; Takahashi et al., 2007b; Lim et al., 2015). It is also noteworthy that expression levels of OCT3/4, SOX2, NANOG, SALL4, E-CADHERIN and hTERT determined by western blotting and were similar in iPSC and hESC (Takahashi et al., 2007b; Yoshida et al., 2017).

In this study we have programed DeepNEU (v3.6) to simulate iPSCs (i.e., aiPSC) using a defined sets of reprograming factors (Danter, 2019) plus MyoD1, a master regulator of skeletal muscle cell differentiation and Doxycycline, a macrolide Tetracycline like antibiotic. Doxycycline is commonly used to facilitate efficient cellular reprograming. The non-antibiotic actions of drugs of the Tetracycline type affect many important cellular pathways (e.g., PI3K-AKT-mTOR pathways) that lead to iPSC and ePSC proliferation and survival (Chang et al., 2014). All simulations in the present study included Doxycycline because they consistently converged faster than without Doxycycline To summarize, we have turned on the key transcription factors that were previously reported to induce pluripotency. Briefly, OCT3/4, SOX2, KLF4 and cMYC were turned on as were MyoD1 and Doxycycline (Takahashi et al., 2007a; Yoshida et al., 2017).

**FIGURE 1:**
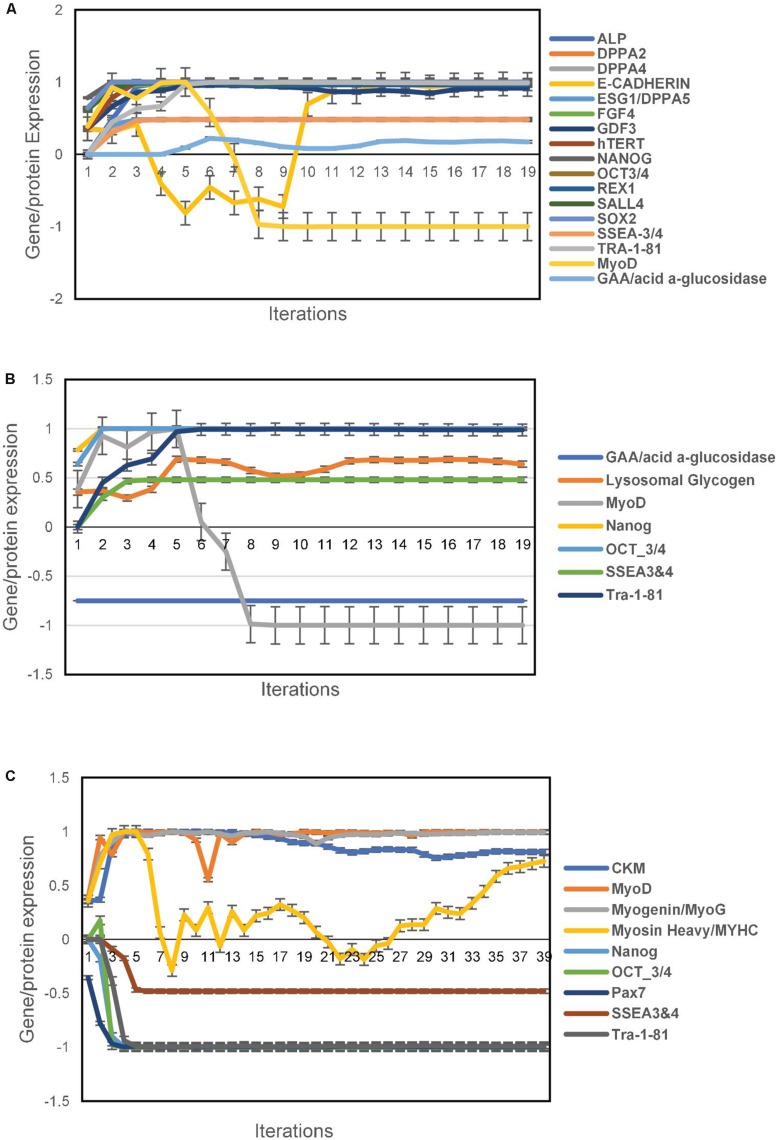
Pluripotency predictions for aiPSC and wild type aiSkMC simulations. **(A)** Gene/protein expression in wild type aiPSC simulation with MyoD1 turned on. **(B)** Gene/protein expression in aiPSC simulation with GAA loss of function mutation. **(C)** Loss of pluripotency in differentiated aiSkMC simulation. Data are representative of three independent simulation experiments; e*rror bars* indicate ± SEM.

The unsupervised aiPSC model converged quickly (19 iterations) to a new system wide steady state without evidence of overtraining after 1000 iterations. The aiPSC model expressed the same human ESC specific surface antigens, including SSEA-3/4, tumor-related antigen TRA-1-81, alkaline phosphatase (ALP) and NANOG protein. Due to inadequate available data the current aiPSC system did not implement the tumor-related antigen TRA-1-60 and therefore it could not be evaluated. Importantly, all the undifferentiated ESC makers mentioned above regarding iPSC were also upregulated/expressed in the aiPSC model system (Figure 1A). Also, in the wild type (WT) aiPSC with MyoD1 turned on there was transient MyoD1 expression without evidence of endogenous/sustained MyoD1 expression consistent with the results obtained by Yoshida et al. (2017). Notably, the WT aiPSC simulations demonstrate that GAA is expressed at levels just above that of normal/baseline. These additional results are also included in Figure 1A. The probability that all (*N* = 17) of these WT aiPSC outcomes were correctly predicted by chance alone using the binomial test is 0.0009.

### The (aiPSC IOPD) Simulation

We next employed DeepNEU to generate the unsupervised aiPSC model of infantile onset POMPE disease (IOPD) by again turning on the transcription factors OCT3/4, KLF4, SOX2, cMYC plus MyoD1 in the presence of Doxycycline, but this time the GAA gene which makes the enzyme normally responsible for lysosomal conversion of glycogen to glucose was locked off. The unsupervised aiPSC IOPD model also converged quickly (19 iterations) to a new system wide steady state without evidence of overtraining after 1000 iterations.

Like the WT aiPSC the IOPD aiPSC also expressed several pluripotency markers including NANOG, OCT3/4, SSEA4 and TRA-1-81 consistent with the results of Yoshida et al. (2017). These data indicate that the MyoD1-transfected aiPSCs retained their pluripotent characteristics in the absence of GAA. The loss of function mutation or deletion of GAA would be expected to result in an increase in lysosomal glycogen, a pathological hallmark of POMPE disease. In fact a modest increase in lysosomal glycogen was observed in the aiPSC IOPD simulations. The probability that these aiPSC IOPD simulation outcomes (*N* = 7) were correctly predicted by chance alone using the binomial test is 0.055. These results are summarized in Figure 1B.

### The aiSkMC (WT) Skeletal Muscle Cell Simulation

Previous research has demonstrated that skeletal muscle cells can be transdifferentiated successfully from human fibroblasts with a single reprograming factor, MyoD1 (Davis et al., 1987; Lattanzi et al., 1998). In these experiments a computer simulation of a human fibroblast was exposed to MyoD1 in the presence of Doxycycline. This means that both factors were turned on as the simulation evolved toward the new steady state. The unsupervised aiSkMC (WT) model converged quickly (39 iterations) to a new system wide steady state without evidence of overtraining after 1000 iterations. Previous research has identified several skeletal muscle markers that confirmed the presence of successfully differentiated/mature skeletal muscle cells (Yoshida et al., 2017). These expressed/up regulated factors included MyoD1, CKM, MyHC, and Myogenin while Pax7 was down regulated. The same pattern of expressed and down regulated markers (*N* = 5) was also produced by the aiSkMC (WT) simulation. In addition, the previously identified pluripotency markers Nanog, Oct3/4, SSE3/4 and Tra-1-81 (*N* = 4) are not present/down regulated in the differentiated aiSkMC (WT) simulation in keeping with a loss of pluripotency. The probability that these markers (*N* = 9) associated with transdifferentiation of fibroblasts (WT) to mature aiSkMC (WT) were accurately predicted by chance alone using the binomial test is 0.024. The results for the aiSkMC (WT) simulation are summarized in Figure 1C.

### The Transdifferentiated Skeletal Muscle Cell Simulation (aiSkMC) of Infantile Onset POMPE Disease (IOPD)

**FIGURE 2:**
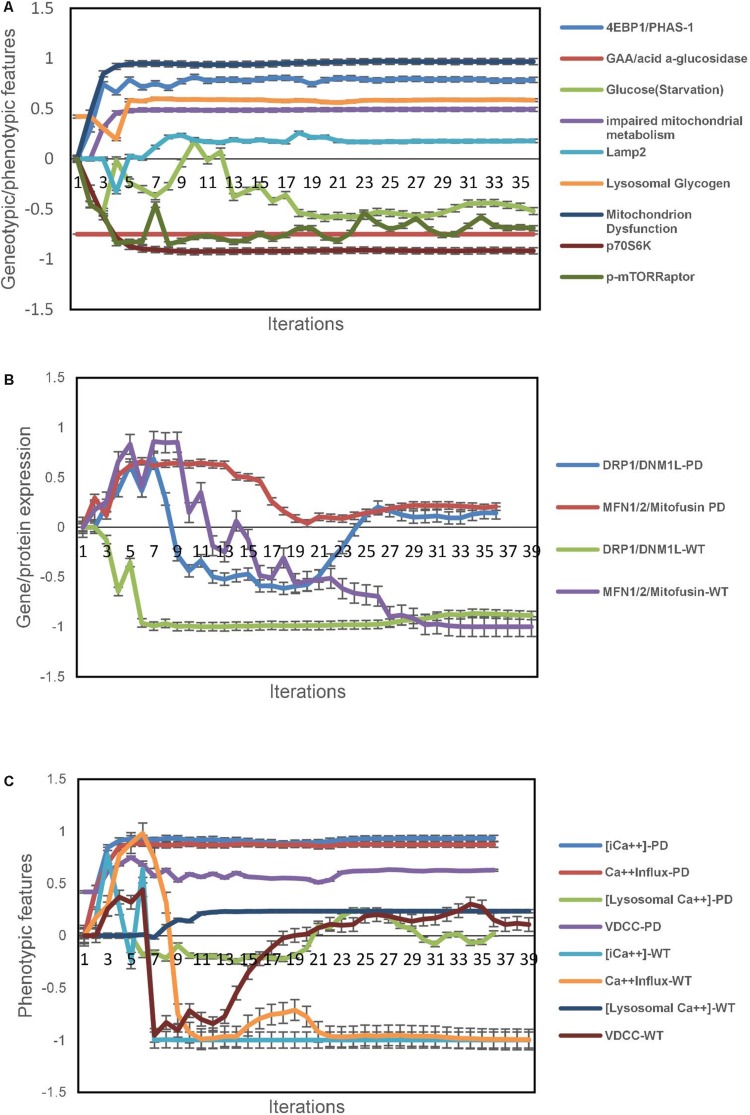
IOPD aiSkMC simulation predictions. **(A)** Gene/protein expression and phenotypic features of aiSkMC IOPD simulation. **(B)** Gene/protein expression of morphological proteins in aiSkMC-IOPD simulation. **(C)** Calcium homeostasis in the IOPD skeletal muscle simulation. Data are representative of three independent simulation experiments; e*rror bars* indicate ± SEM.

Next, we used the unsupervised aiSkMC model that was validated above with the gene GAA locked off to simulate aiSkMC IOPD. The model converged quickly (36 iterations) to a new system wide steady state without evidence of overtraining after 1000 iterations. The up and down regulated marker profile (*N* = 9) identified for the aiSkMC (WT) simulation was also reproduced by the aiSkMC IOPD simulation. Importantly, a hallmark of IOPD is the marked accumulation of lysosomal glycogen. While the undifferentiated aiPSC model with GAA deletion demonstrated a modest increase in lysosomal glycogen the differentiated aiSkMC simulations of IOPD revealed a highly significant increase in lysosomal glycogen (mean ± 99%CI = 0.585 ± 0.003) when compared with the aiSkMC WT simulations (mean ± 99%CI = −0.297 ± 0.039; Mann–Whitney *u* test exact *p*-value = 1.29E-08). In addition, GAA expression in the setting of glucose deprivation was markedly decreased as expected while lysosomal glycogen and LAMP2 were increased/upregulated (Figure 2A).

Also, the same study (Yoshida et al., 2017) carried out a detailed evaluation of mTORC1 activation and energy metabolism in Pompe disease iPSC-derived myocytes. First, the study demonstrated that mTORC1 activity was suppressed in association with lysosomal glycogen accumulation as was the phosphorylation of two important downstream targets of mTORC1 namely p70S6K and p4EBP1. Then they employed several indices including adenylate energy charge, guanylate energy charge, NAD+/NADH ratio and glucose 6-phosphate/ribose 5-phosphate ratio to conclude that energy metabolism was impaired in Pompe disease myocytes and that the metabolic impairment was at least in part related to mitochondrial dysfunction. These wet lab results indicating impaired mitochondrial energy metabolism were accurately predicted by the aiSkMC IOPD simulation (Figure 2A). mTORC1 phosphoactivation is significantly suppressed in the simulations as is the phosphorylation of downstream targets p70S6K and p4EBP1. Importantly, mitochondrial dysfunction was also significantly increased in the aiSkMC IOPD simulation. The results are also summarized in Figure 2A. Mitochondrial dysfunction is an important and complex phenotypic concept in DeepNEU 3.6 simulations having 77 gene/protein or phenotypic inputs and 40 gene/protein or phenotypic outputs. The probability that these genetic and phenotypic markers (*N* = 9) associated with the aiSkMC IOPD simulations were accurately predicted by chance alone using the binomial test is 0.024.

### Application of the Validated aiSkMC Simulation to Disease Modeling, Biomarker Identification and Drug Discovery

The central role of abnormal calcium homeostasis and mitochondrial dysfunction in POMPE disease has been studied in detail by Lim et al. (2015) using an *in vitro* skeletal muscle cell model with the GAA gene deleted. Complete gene deletion of GAA would be expected to most closely represent the more severe, infantile form of POMPE disease (IOPD). We chose to use this peer reviewed paper to validate the aiSkMC simulation because it addresses elements of disease modeling, biomarker identification and drug discovery. We therefore evaluated aiSkMC IOPD predictions regarding (1) mitochondrial morphology, (2) calcium homeostasis, (3) mitochondrial physiology and function, (4) mitochondrial bioenergetics, (5) autophagy and mitophagy (6) potential restoration of more normal calcium homeostasis and (7) finally we used the data generated by the aiSkMC to extend the findings of Lim et al. (2015).

Considering the current version of DeepNEU (v.3.6), data on 3871 gene/protein/phenotypic features were available for analysis (PubMed/MedLIne as per Danter, 2019). The analysis involved a comparison of WT aiSkMC vs. aiSkMC IOPD based on the specific genotypic/protein/phenotypic features reported by Lim et al. (2015). Initial statistical analysis of the data used the unpaired *t*-test with unequal variances to identify high level differences between WT and IOPD simulations. For the purposes of this paper we focused on calcium homeostatic and mitochondrial factors wherever possible.

#### Morphological Changes of Mitochondria in Pompe Disease Muscle Cells

Lim et al. (2015) used a combination of transmission electron microscopy, various enzymatic dye assays and Western blotting to evaluate mitochondrial structure. In this analysis several “shaping proteins” were elevated in POMPE muscle cells. These proteins including DNM1L, FIS1, OPA1, MFN2 and COX4l1 were elevated. While the current version of DeepNEU (v3.6) does not include direct measures of mitochondrial morphology, two of the five “shaping proteins” were previously included and could be evaluated. Both DNM1L and MFN2 were expressed/overexpressed in the aiSkMC-IOPD simulation compared to the WT. These data are presented in Figure 2B.

#### Calcium Homeostasis Is Deranged in Pompe Disease Muscle Cells

The same study mentioned above used multiple techniques to investigate elements of calcium homeostasis using their *in vitro* POMPE skeletal muscle model (Lim et al., 2015). These authors identified a marked increase in intracellular calcium concentration. They then identified that this increase was the result of calcium influx via active calcium channels in POMPE skeletal muscle cells compared to WT. Cellular staining suggested that while intracellular calcium did not selectively accumulate in lysosomes there was calcium accumulation/overload in POMPE cell mitochondria.

Data from the aiSkMC IOPD simulation confirmed the elevated intracellular calcium concentration and that this increase was related to an increase in calcium influx via calcium channels of the VDCC class. Lysosomal calcium concentration was not different from WT. Unfortunately, mitochondrial calcium concentration was not implemented in this version of DeepNEU and therefore could not be evaluated. These data are summarized in Figure 2C. All the available factors (*N* = 4) predicted by the aiSkMC IOPD simulation are entirely consistent with deranged calcium homeostasis as identified in the *in vitro* cellular model.

#### Mitochondrial Physiology and Function Are Altered in Pompe Disease Muscle Cells

**FIGURE 3:**
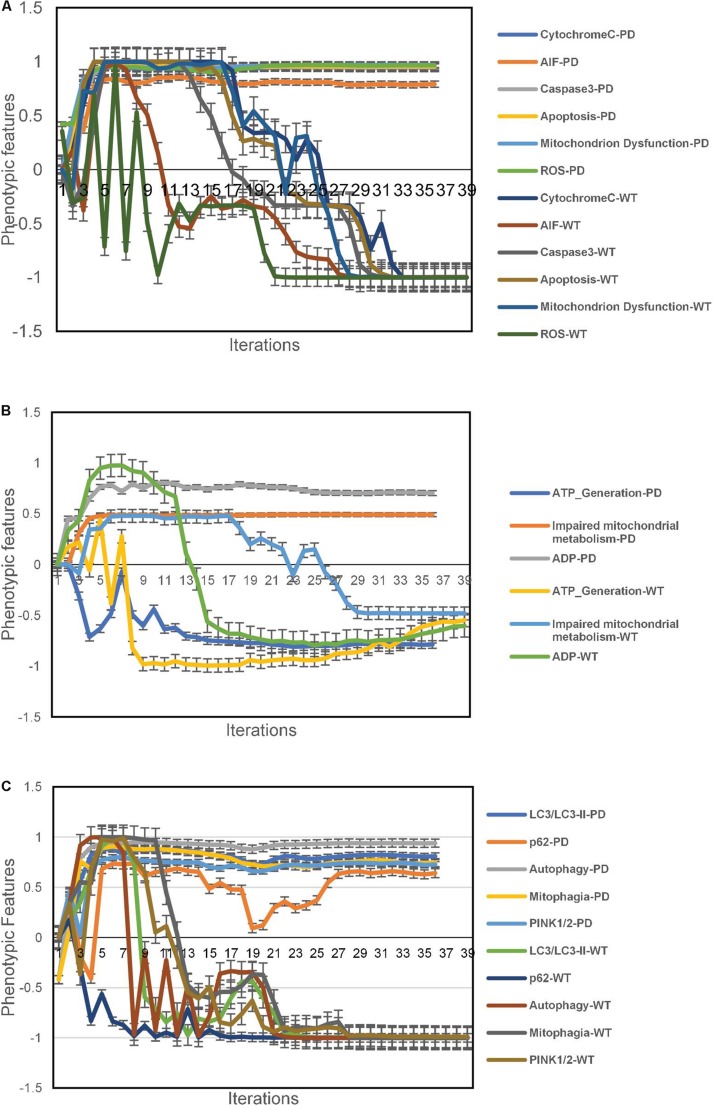
Phenotypic feature predictions for IOPD vs. WT aiSkMC simulations. **(A)** altered mitochondrial physiology and function in aiSkMC simulations. **(B)** Defective mitochondrial bioenergetics in IOPD SkMC simulations. **(C)** Defective autophagy and mitophagy in IOPD aiSkMC simulations. Data are representative of three independent simulation experiments; e*rror bars* indicate ± SEM.

The aiSkMC IOPD simulations produced data consistent with deranged mitochondrial physiology and function. The mitochondrial dysfunction was associated with increased Cytochrome C, AIF and ROS. Caspase 3 levels were also elevated and there was evidence of increased apoptosis. These data are summarized in Figure 3A.

#### Mitochondrial Bioenergetics Is Defective in Pompe Disease Muscle Cells

The aiSkMC IOPD simulation also provided evidence of defective mitochondrial bioenergetics consistent with the wet lab results reported by Lim et al. (2015). First, ADP levels were markedly elevated compared with WT. Consistent with the increased levels of ADP there was also a reduction ATP levels (i.e., ATP generation) compared with WT. Finally, the aiSkMC IOPD simulation predicted that mitochondrial metabolism was indeed impaired as a subnetwork of mitochondrial dysfunction. These results are summarized in Figure 3B.

#### Autophagy and Mitophagy Are Affected in Pompe Disease Muscle Cells

The aiSkMC IOPD simulation predicted that autophagy and mitophagy are active in POMPE disease (IOPD). Levels of LC3/LCII specific markers for autophagosomes are significantly elevated in IOPD. Also, p62(SQSTM1) a receptor for LC3 is elevated in IOPD indicating that autophagy is likely to be incomplete in diseased skeletal muscle cells. Regarding mitophagy, the study (Lim et al., 2015) identified increased Pink2 and Park2 levels indicating active mitophagy in POMPE muscle cells. While Park2 is not implemented in the current version of DeepNEU (v3.6) Pink2 was previously included and was found to be significantly increased in the aiSkMC simulation. All the implemented factors (*N* = 5) were consistent with the results reported before (Lim et al., 2015) and are summarized in Figure 3C.

#### More Normal Calcium Homeostasis Can Be Restored in Pompe Disease Muscle Cells

**FIGURE 4:**
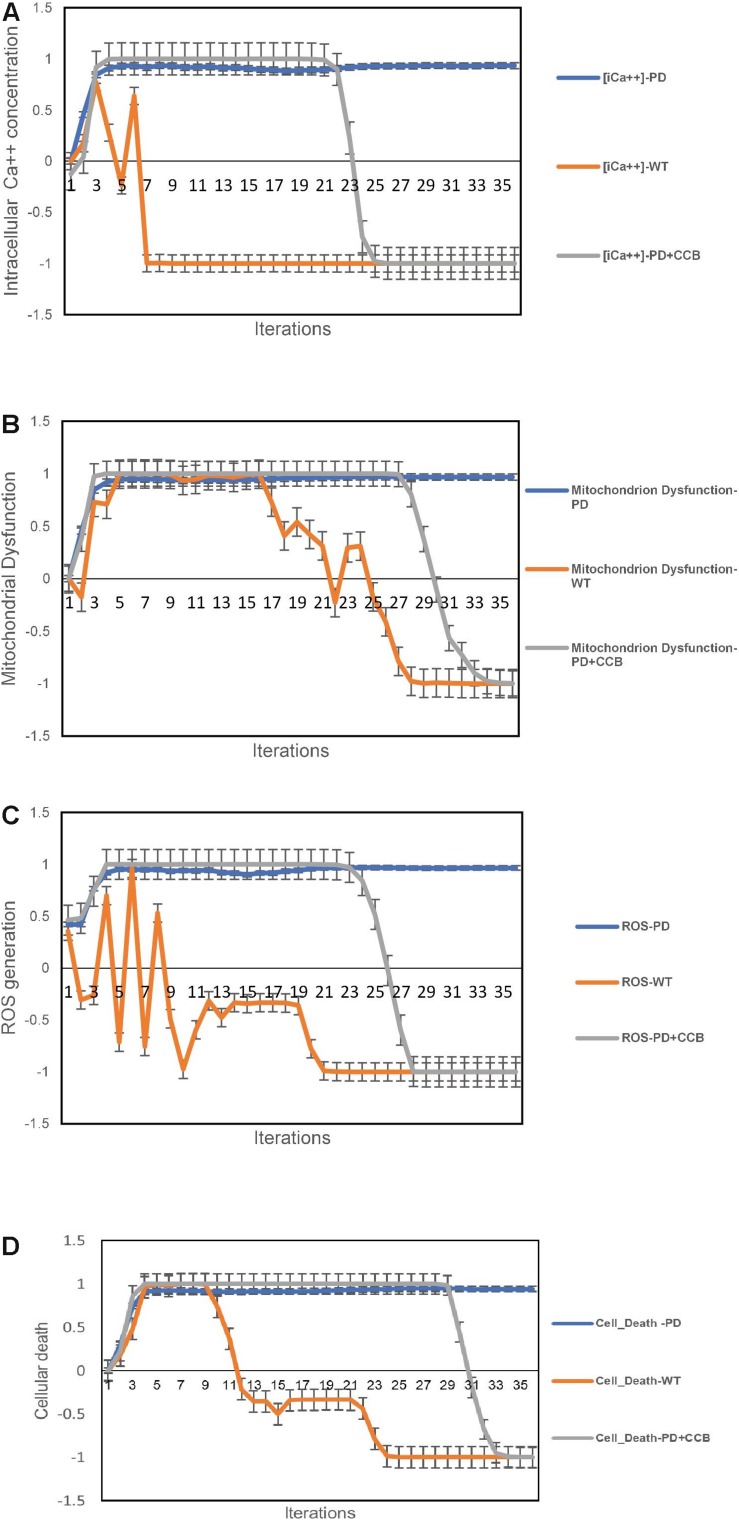
Predicted effects of calcium channel blockade (CCB) (IOPD vs. WT simulations). **(A)** Effect of CCB on intracellular calcium concentration. **(B)** Effect of CCB on mitochondrial dysfunction. **(C)** Effect of CCB on Reactive Oxygen Species (ROS). **(D)** Effect of CCB on cell death. Data are representative of three independent simulation experiments; e*rror bars* indicate ± SEM.

In the previous study, the authors successfully established and characterized features of a GAA knock out *in vitro* skeletal muscle model of POMPE IOPD (Lim et al., 2015). Once the model was characterized these authors went on to show that the observed abnormal calcium homeostasis and mitochondrial dysfunction could be improved by treatment with a calcium channel blocker (CCB). They specifically evaluated the effects of the CCB on intracellular calcium and ROS. The aiSkMC IOPD simulation with VDCC locked off to simulate CCB was used to determine whether the simulation could reproduce similar results with respect to intracellular calcium, ROS, mitochondrial dysfunction and cell death. All doses of CCB producing at least 25% VDCC inhibition (i.e., 25, 50, 75, and 100%) produced marked improvement in all parameters. These data suggest that even modest inhibition of VDCC produces significant improvements in intracellular calcium, ROS, mitochondrial dysfunction and cell death. The results at 25% VDCC inhibition are entirely consistent with the data provided by Lim et al. (2015) and are summarized in Figures 4A–D.

#### DeepNEU SkMC Simulation Identifies Calpain(s) as a Potential Therapeutic Target for IOPD

While comparing aiSkMC predictions for IOPD vs. WT simulations and focusing on a comprehensive subset of factors related to calcium homeostasis, calpain(s) stood out as a potential biomarker. Based on the two tailed Mann–Whitney *u* test, the estimated *p*-value for aiSkMC IOPD vs. WT was highly significant at <0.0001. To further explore this finding a dataset (*N* = 75) of IOPD and WT profiles was created. These data were modeled using validated gene expression programing (GEP) software (DTREG version 10.7.18, 2016). A single variable equation i.e., OUTCOME{Normal} = (−42.297 ^∗^ calpain) + 10.791 was evolved. Tenfold cross validation produced a classification model (IOPD vs. WT) with the following operator characteristic profile: Precision = 94.59%, Recall = 97.22%, F-Measure = 0.959 and Area under ROC curve (AUC) = 0.973. Based on these results calpain(s) was confirmed as a potential biomarker and therapeutic target for IOPD warranting further evaluation.

**FIGURE 5:**
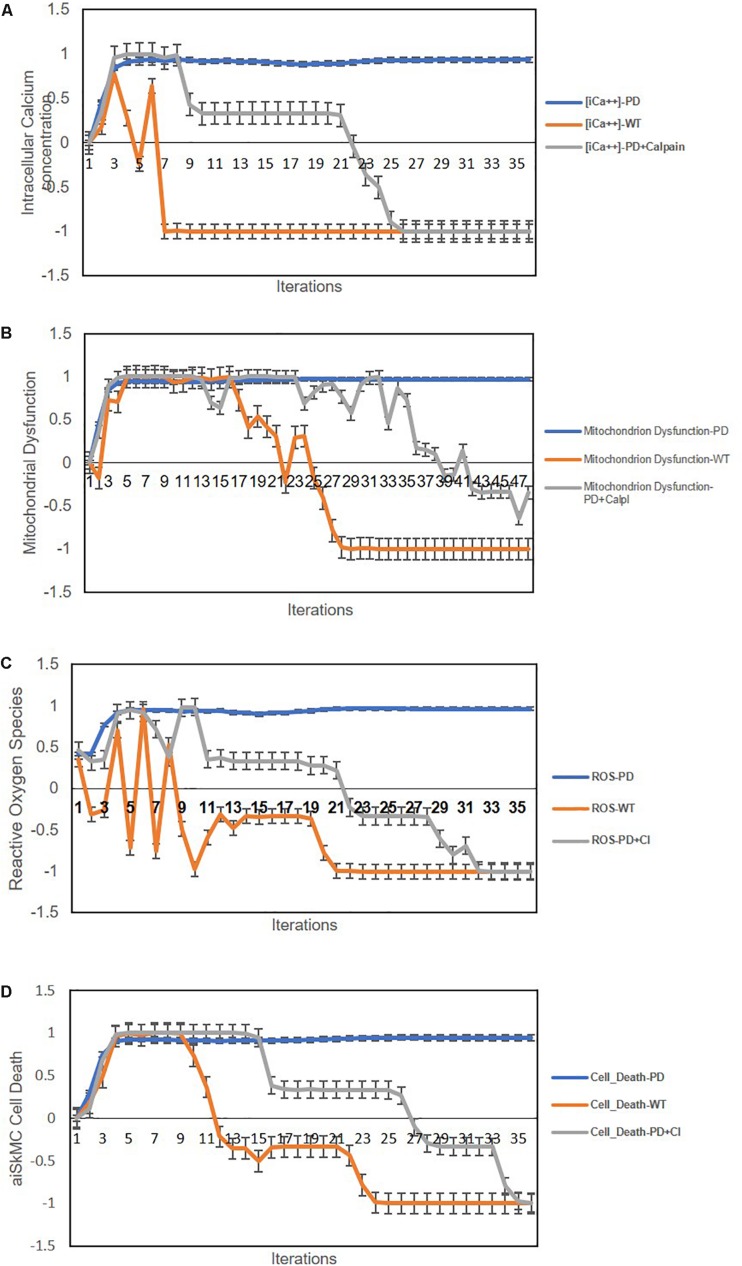
Predicted cellular effects of limited (i.e., ≤20% of maximum) calpain Inhibition (IOPD vs. WT simulations). **(A)** Effect of calpain inhibition on intracellular calcium concentration. **(B)** Calpain inhibition effect on mitochondrial dysfunction. **(C)** Effect of calpain(s) inhibition on ROS. **(D)** Effect of calpain(s) inhibition on cell death. Data are representative of three independent simulation experiments; e*rror bars* indicate ± SEM.

We next used the aiSkMC IOPD simulation with calpain(s) variably locked off between 0 and −1 to simulate calpain inhibition, to investigate whether the simulation would produce similar results with respect to intracellular calcium, ROS, mitochondrial dysfunction and cell death as observed with CCB. All levels of calpain inhibition of at least 10% (i.e., 10, 15, 20, 25, 50, 75, and 100%) produced marked improvement in all phenotypic features. These data suggest that even modest inhibition of calpain(s) (i.e., 10% or greater) produces significant improvements in intracellular calcium, ROS, mitochondrial dysfunction and cell death, like the results observed for CCB. It is important to note that inhibition levels less than 20% did not prevent the establishment of the disease phenotype prior to reversal to a more normal state. These results are summarized in Figures 5A–D. Summary of the main findings are presented below in Table 2.

**TABLE 2:** Summary of DeepNEU IOPD simulation main findings.

**DeepNEU Predictions (v3.6)**	**State**	**WT**	**Binomial Test**	**IOPD**	**Binomial Test**	**Validation**
		**Features (N)**	**(WT, 2 tailed)**	**Features (N)**	**(IOPD, 2 tailed)**	
The aiPSC (WT) simulations	Undifferentiated	17	0.0009	0	N/A	Literature
The aiPSC (IOPD) simulations	Undifferentiated	17	0.0009	7	0.055	Literature
The aiSkMC (WT) simulations	Differentiated	9	0.024	0	N/A	Literature
The aiSkMC (IOPD) simulations	Differentiated	9	0.024	9	0.024	Literature
**Application of the validated aiSkMC IOPD simulations**						
Morphological changes of mitochondria	Differentiated	NA	NA	2	NA	Literature
Deranged Calcium homeostasis	Differentiated	NA	NA	4	NA	Literature
Altered Mitochondrial physiology and function	Differentiated	NA	NA	5	NA	Literature
Defective Mitochondrial bioenergetics	Differentiated	NA	NA	3	NA	Literature
Altered autophagy and mitophagy	Differentiated	NA	NA	5	NA	Literature
More normal calcium homeostasis can be restored	Differentiated	NA	NA	4	NA	Literature
**Total features**		NA	NA	23	0.00008	Literature
**Potential Biomarker for IOPD**						
Calpain(s) identified as a potential therapeutic target	Differentiated	0.893 ± 0.0003	0.999 ± 0.000002	10	<0.0001	WetLab/TBD

## Discussion

The main purpose of this research was to generate and then validate potentially useful computer simulations of infantile onset Pompei disease (IOPD). The data from these experiments indicate that IOPD at the cellular level can indeed be accurately simulated in both aiPSC and aiSkMC using the DeepNEU (v3.6) AI platform. We believe that, as of this writing, these are the first and only results of this kind in the published literature. Importantly, these results are consistent with and extend our earlier research.

In this regard the current aiPSC (WT) simulation results are remarkably consistent with results from the WT aiPSC models previously reported in Danter (2019). The new simulations also (1) rapidly achieved a new system wide steady state, (2) showed no evidence of overtraining to 1000 iterations and (3) can accurately reproduce wet lab results from the recent peer reviewed literature (Takahashi et al., 2007b) based on the bias adjusted binomial test.

The aiPSC (IOPD) simulations were created by adding the simulated deletion of the GAA gene to the aiPSC (WT) initial input vector and allowing the system to evolve to a new stable steady state. The gene deletion was created by locking off the GAA concept. This ensures that the complete loss of GAA is propagated through the relationship matrix from iteration to iteration consistent with the way that the gene deletion would be transmitted from generation to generation in affected living cells. Importantly, the aiPSC carrying the simulated gene deletion were also able to accurately reproduce the genotypic and phenotypic features of wet lab affected stem cells without loss of pluripotency. For example, the aiPSC IOPD simulation accurately predicted a modest increase in lysosomal glycogen which is entirely consistent with the “small amount of glycogen accumulated” in undifferentiated IOPD iPSC lysosomes (Yoshida et al., 2017). The literature validated aiPSC simulations of IOPD represent a cost-effective tool for early stage disease modeling and experimental rapid prototyping.

To more accurately simulate IOPD we next created aiSkMC by exposing the basic DeepNEU representation of a human fibroblast to MyoD1 and Doxycycline. This direct generation of skeletal muscle cells from human fibroblasts is well documented in the peer reviewed literature (Samuel, 1967; Takahashi et al., 2007a). Using the same approach, the simulation evolved quickly to a new steady state that accurately reproduced the genotypic and phenotypic features of differentiated wild type human skeletal muscle cells (aiSkMC WT). We then created the aiSkMC (IOPD) simulations by adding the simulated deletion of the GAA gene to the aiSkMC (WT) initial input vector and allowing the system to evolve to a new stable steady state. As seen in the aiSkMC (IOPD) simulations the genotypic and phenotypic features of differentiated human muscle cells were preserved while the aiSkMC (IOPD) simulations. also reproduced the genotypic and phenotypic features of IOPD. For example, the aiSkMC IOPD simulations accurately predicted a hallmark of IOPD, namely a marked increase in lysosomal glycogen (exact *p* = 1.29E-08) compared with the aiSkMC WT simulations. Importantly, the generic aiSkMC simulations can also be used to investigate other genetic diseases affecting skeletal muscle like Duchenne and Becker muscular dystrophies.

Once the WT and IOPD aiSkMC simulations were created and validated against the available peer reviewed wet lab research, they were applied to additional specific areas including disease modeling, biomarker identification and drug discovery.

Lim et al. (2015) found that five “shaping proteins” were elevated in POMPE muscle cells. These proteins included DNM1L, FIS1, OPA1, MFN2, and COX4l1. The elevation of these specific proteins is consistent with abnormal mitochondrial morphology. Two of the five “shaping proteins” are included in the current version of DeepNEU and both DNM1L and MFN2 were expressed/overexpressed in the aiSkMC-IOPD simulation compared to the WT. While no firm conclusions about mitochondrial morphology can be based on these limited data (*N* = 2), the elevated DNM1L and MFN2 protein levels observed for the aiSkMC IOPD simulation are at least consistent with altered mitochondrial morphology observed in POMPE IOPD. DeepNEU can be easily updated and future versions could include FIS1, OPA1 and COX4l1 specific pathways to further evaluate mitochondrial morphology in the aiSkMC IOPD simulations.

The same authors used multiple techniques to confirm that calcium homeostasis is deranged in POMPE muscle cells. The abnormalities identified included (1) a marked increase in intracellular calcium concentration, (2) increased calcium influx via active calcium channels and (3) calcium selectively accumulated in POMPE muscle cell mitochondria but not in lysosomes. The aiSkMC IOPD simulation experiments confirmed the elevated intracellular calcium concentration and that this increase was related to an increase in calcium influx via calcium channels of the VDCC class. Lysosomal calcium concentration was not different from WT. The concept of mitochondrial calcium concentration was not implemented in this version of DeepNEU and therefore could not be evaluated. All the available factors (*N* = 4) predicted by the aiSkMC IOPD simulation are entirely consistent with deranged calcium homeostasis as identified in the *in vitro* cellular model (Lim et al., 2015). Future versions of DeepNEU will include concepts specifically related to mitochondrial calcium homeostasis.

The aiSkMC IOPD simulation experiments also produced data consistent with mitochondrial dysfunction. This dysfunction was associated with increased Cytochrome C, AIF, ROS, Caspase 3 levels and apoptosis. These data are entirely consistent with the findings of Lim et al. (2015). While these authors did not find a significant difference in Caspase 3 levels between POMPE and WT muscle cells, both the aiSkMC IOPD and POMPE *in vitro* models did show an increase in apoptosis with the aiSkMC IOPD simulation result being more pronounced than the *in vitro* POMPE model. Arguably all six features of deranged mitochondrial physiology and function were predicted correctly by the aiSkMC IOPD simulation.

Mitochondria are the primary energy generating cellular organelles and the aiSkMC IOPD simulation provided evidence of defective mitochondrial bioenergetics consistent with the *in vitro* results reported by Lim et al. (2015). First, ADP levels were markedly elevated compared with WT consistent with the mitochondria being in state III, typical of ADP driven respiration associated with decreased mitochondrial oxygen consumption. Also consistent with decreased mitochondrial oxygen consumption is reduced ATP generation. Finally, the aiSkMC IOPD simulation predicted that mitochondrial metabolism was indeed impaired as a relatively large subnetwork of mitochondrial dysfunction. Taken together the data indicate that the aiSkMC IOPD simulations can accurately reproduce the abnormalities in mitochondrial physiology, function and bioenergetics found in wet lab experiments and discussed in Lim et al. (2015).

Based on the aiSkMC IOPD simulations both autophagy and mitophagy are active and deranged in POMPE disease (IOPD). Levels of LC3/LCII, specific markers for autophagosomes, are significantly elevated in IOPD. Also, a receptor for LC3 [i.e., p62(SQSTM1)] is elevated in IOPD indicating that while active, autophagy is probably incomplete in diseased skeletal muscle cells. Lim et al. (2015) also identified increased Pink2 and Park2 levels indicating active mitophagy in POMPE muscle cells. While Park2 is not implemented in the current version of DeepNEU (v3.6) Pink2 was included and found to be significantly increased in the aiSkMC simulation. All the implemented factors (*N* = 5) were correct and consistent with the results reported previously (Lim et al., 2015).

To determine if the observed abnormalities of calcium homeostasis and mitochondrial function observed in POMPE muscle cells could be improved by specific treatment, study demonstrated that the use of a CCB could restore a more normal intracellular calcium concentration, ameliorate ROS and improve mitochondrial function in the *in vitro* disease model (Lim et al., 2015). The aiSkMC IOPD simulation with VDCC locked off to simulate CCB was used to determine whether the simulation could produce similar results with respect to intracellular calcium, ROS and mitochondrial dysfunction. Different fractional doses of the CCB was simulated by altering the amount of VDCC inhibition between 0 and 100%. All doses of CCB producing at least 25% VDCC inhibition (i.e., 25, 50, 75, and 100%) produced marked improvement in all parameters after the initial establishment of the disease phenotype. These data suggest that even modest inhibition of VDCC produces significant improvements in intracellular calcium, ROS and mitochondrial function and cell death. The results at 25% VDCC inhibition are entirely consistent with the CCB data provided in Lim et al. (2015). Notably, these results also demonstrate the ease with which DeepNEU can represent fractional relationships.

In addition to disease modeling DeepNEU has also been designed to be used as a tool for rapid and cost-effective biomarker and drug discovery. The use of stem cells for targeted drug discovery has been well reported in the peer reviewed literature (Young et al., 2012; Elitt et al., 2018; Han et al., 2018). Our approach to this issue has three simple components. First, we compared the aiSkMC predictions from the IOPD vs. WT simulations, focusing on the comprehensive subset of factors involved in calcium homeostasis. The statistical analysis identified calpain(s) as an important potential biomarker. We used the Mann-Whitney test to estimate the level of significance. The *p*-value for aiSkMC IOPD vs. WT was highly significant at <0.001. Second, to further explore this finding, a dataset (*N* = 75) of IOPD and WT calpain profiles was created. These data were modeled using validated gene expression programing (GEP) software (DTREG version 10.7.18, 2016). A single variable equation i.e., OUTCOME{Normal} = (−42.297 ^∗^ calpain) + 10.791 was evolved. Tenfold cross validation produced a classification model with the following operator characteristic profile: Precision = 94.59%, Recall = 97.22%, F-Measure = 0.959 and Area under ROC curve (AUC) = 0.973. Based on the combined results calpain(s) was confirmed as a potential biomarker and therapeutic target for IOPD warranting further evaluation.

Next, we went on to use the validated aiSkMC IOPD simulation with calpain(s) variably locked off to further explore its’ therapeutic potential. The aiSkMC IOPD simulation with calpain(s) locked off to simulate calpain inhibition was used to determine whether the simulation would produce similar results with respect to intracellular calcium, ROS, mitochondrial dysfunction and cell death observed with CCB. In fact, all levels of calpain inhibition of at least 10% produced a marked improvement in all parameters. These data indicate that even modest inhibition of calpain(s) (i.e., 10%) produces clear improvements in intracellular calcium, ROS, mitochondrial dysfunction and cell survival, like the results observed for CCB. We also observed that at calpain inhibition greater than 20% all disease phenotypic factors declined rapidly toward zero even before the disease phenotype is established. These simulated results are difficult to interpret but could suggest that calpain is a critical factor in the very earliest development of the IOPD phenotype. As of this writing we are not aware of any literature specifically identifying calpain(s) as a potential biomarker or therapeutic target specific to IOPD.

### Current DeepNEU Limitations

#### Incomplete Data

All models, no matter how sophisticated require, as a minimum, adequate representative data of the subject to be modeled. In the case of human stems cells our current scientific knowledge is incomplete but continues to grow rapidly. This lack of complete representative data results in the major limitation of the DeepNEU platform. While we add new gene and protein relationship data daily the current database contains relationships involving approximately 15% of the human genome. Put another way, about 85% of the gene-gene and gene-protein relationships are not represented or remain unknown. A more positive assessment is that a critical mass of data is required to produce meaningful models and that these models will become more useful over time. Anecdotally, the prediction accuracy of DeepNEU has steadily increased and the evolution of stable models requires fewer iterations as the number of concepts and relationships has increased.

#### Wet Lab Validation

Currently, predictions from advanced computer modeling systems still require wet lab confirmation and DeepNEU is no exception. One of the original goals of this research was to make the information regarding the potential therapeutic use of calpain(s) inhibitors in IOPD freely available to the global rare disease research community for wet lab validation. However, we did plan and still plan to validate these predictions with our own wet lab experiments, and we are currently looking for development partners with the goal of confirming the role of calpain inhibitors with and without CCB in an animal model of IOPD. We plan on making this additional information available at the earliest opportunity.

#### Software and Hardware

Useful models also require effective software and computer hardware. Fortunately, non-linear high dimensional modeling software is widely available and evolving rapidly. The current DeepNEU platform combines multiple algorithms including elements of advanced artificial neural networks, cognitive maps, support vector machines and evolutionary systems. DeepNEU also relies exclusively on an unsupervised learning approach and makes limited if any assumptions about the data to be modeled. We believe that this approach which is referred to as stochastic or near assumption free and unsupervised learning using hybrid learning systems represents the current state of the art in the area of machine learning. This is likely to change over time. Available hardware also appears adequate for the demands of high dimension non-linear modeling. High end laptop computers for example, have fast multiple core processors, multi-gigabyte onboard memory, terabyte solid state hard drives, 64/128-bit operating systems and large companies like IBM will soon be introducing quantum computing for the general consumer.

#### Deep Learning

Finally, the current state of ML with its reliance on Deep Learning may itself also impose some limitations on the potential of the DeepNEU platform. Another relatively new approach to machine learning (ML) called Wise Learning (WL) will at least compliment or eventually even supplant the current DL approach. Wise Learning has been defined by Groumpos (2016) as “having or showing the ability to make true, right, lasting, prudent, proper and good judgments, based on a deep understanding and experience of nature and life, and above all to be beneficial to humankind.” The same author has also provided a detailed and useful comparison of the strengths and weaknesses of DL vs. WL. DeepNEU (v. 3.6) has purposely incorporated basic elements of Wise Learning. These include relying on multiple expert sources of data and combining cognitive mapping with Deep Learning technologies. As DeepNEU continues to evolve the goal is to continue the transition to a WL based process. It is inevitable that WL itself will over time be replaced by a more advanced process.

#### DeepNEU and Calpain(s) as Potential Disease Modifier(s)

The exact role of calpain(s) in IOPD remains unclear. While they may indeed be biomarkers and therefore potential drug targets, there may be at least one other potential and important explanation. Calpain(s) could be disease modifier(s) in IOPD and perhaps Pompe disease in general. A recent review of calpains in health and disease points out that calpain(s) clearly play a role as disease modifiers in neurodegeneration and Huntington’s Disease in particular. If this turns out to be the case then the current version of DeepNEU cannot distinguish between biomarkers and disease modifiers. Fortunately both possibilities are important and can be resolved through the process of wet lab validation (Weber et al., 2019).

Overall, we have demonstrated that the DeepNEU (v. 3.6) derived aiSkMC simulations of IOPD are robust and can be used for disease modeling, potential biomarker identification and could lead to targeted drug discovery. Furthermore, based on these results we are continuing to explore the potential for calpain inhibitors with and without CCB as effective therapy for IOPD.

## Conclusion/Significance

We expect that machine learning (ML) technologies will continue to transform stem cell research. The results of our continued DeepNEU research confirm that currently available stem cell data, computer software and hardware are adequate to generate basic artificially induced pluripotent stem cells (aiPSC) as well as specific transdifferentiated cell line specific disease models. The present DeepNEU (v. 3.6) simulations of infantile onset POMPE disease (IOPD) have accurately reproduced gene/protein and phenotypic results from the only peer reviewed iPSC and skeletal muscle cell models of IOPD (Lim et al., 2015). The validated disease models of IOPD represent a potentially transformative new technology to empower rare disease researchers.

The application of this computer technology to generate multiple disease specific aiPSCs and tissue types has the potential to improve (1) disease modeling, (2) rapid prototyping of wet lab experiments, (3) grant application writing and (4) specific biomarker identification and targeted drug discovery all in a cost-effective manner. Continued development and validation of this promising new technology is ongoing with the primary focus on modeling other rare genetic diseases in addition to IOPD.

## Data Availability Statement

The datasets generated for this study are available on request to the corresponding author.

## Author Contributions

SE and WD conceptualized, planned, and analyzed the experimental work, wrote the manuscript, and prepared the figures. WD performed all computational simulations and disease modeling.

## Conflict of Interest

SE and WD were both employed by 123Genetix.

## Footnotes

1 https://ghr.nlm.nih.gov/condition/pompe-disease
